# Seasonal climate effects on the survival of a hibernating mammal

**DOI:** 10.1002/ece3.5000

**Published:** 2019-02-27

**Authors:** Caylee A. Falvo, David N. Koons, Lise M. Aubry

**Affiliations:** ^1^ Department of Fish, Wildlife, and Conservation Biology Colorado State University Fort Collins Colorado; ^2^ Graduate Degree Program in Ecology Colorado State University Fort Collins Colorado

**Keywords:** capture–mark–recapture, climate change, fitness, ground squirrel, phenology, survival

## Abstract

Global climate change and associated regional climate variability is impacting the phenology of many species, ultimately altering individual fitness and population dynamics. Yet, few studies have considered the effects of pertinent seasonal climate variability on phenology and fitness. Hibernators may be particularly susceptible to changes in seasonal climate since they have a relatively short active season in which to reproduce and gain enough mass to survive the following winter. To understand whether and how seasonal climate variability may be affecting hibernator fitness, we estimated survival from historical (1964–1968) and contemporary (2014–2017) mark–recapture data collected from the same population of Uinta ground squirrels (UGS, *Urocitellus armatus*), a hibernator endemic to the western United States. Despite a locally warming climate, the phenology of UGS did not change over time, yet season‐specific climate variables were important in regulating survival rates. Specifically, older age classes experienced lower survival when winters or the following springs were warm, while juveniles benefited from warmer winter temperatures. Although metabolic costs decrease with decreasing temperature in the hibernacula, arousal costs increase with decreasing temperature. Our results suggest that this trade‐off is experienced differently by immature and mature individuals. We also observed an increase in population density during that time period, suggesting resources are less limited today than they used to be. Cheatgrass is now dominating the study site and may provide a better food source to UGS than native plants did historically.

## 1 INTRODUCTION

Climate change is impacting the phenology of many species, ultimately altering their fitness and population dynamics (Parmesan, 2006; Visser & Both, 2005). For instance, earlier snowmelt in montane habitats causes wildflowers to germinate earlier, increasing their susceptibility to mid‐June frost‐kills, ultimately leading to a reduction in recruitment by phenological mismatch (Inouye, 2008). Shifts in phenology have been documented across a variety of taxa and ecosystems, although the direction of these shifts is inconsistent (Kharouba et al., 2018; Parmesan & Yohe, 2003). Phenological mismatches can occur when the phenology of a given organism does not line up with that of relevant abiotic factors, food resources, conspecifics, or predators, complicating our ability to understand how phenological shifts may affect the focal organism's fitness (Miller‐Rushing, Høye, Inouye, & Post, 2010). In a long‐term study of great tits (*Parus major*) in the Netherlands, disparities in the phenological response to climate change across trophic levels have increased the asynchrony between great tit hatching date and peak caterpillar biomass (Visser, van Noordwijk, Tinbergen, & Lessells, 1998), the preferred source of food for nestlings, which has decreased fledging success (Visser, Holleman, & Gienapp, 2006). However, in a long‐term study of great tits in Wytham Woods, England, the birds have shifted their phenology in response to warming springs while managing to maintain synchrony of their hatch date with peak food abundance by altering their incubation period (Cresswell & McCleery, 2003; Reed, Jenouvrier, & Visser, 2013). These studies illustrate how difficult it can be to predict demographic consequences of phenology shifts, particularly because linking phenology and demography requires extensive and individually based data that are often unavailable (Miller‐Rushing et al., 2010; Plard et al., 2014). Although there are several studies that have directly quantified the effects of phenological shifts on fitness components (Arlt & Pärt, 2017; Møller, Rubolini, & Lehikoinen, 2008; Saino et al., 2011), only a few pertain to mammals (Lane, Kruuk, Charmantier, Murie, & Dobson, 2012; Ozgul et al., 2010).

Hibernating mammals may be particularly susceptible to changes in climate through phenological shifts because they have a relatively short active season in which to mate and gain enough weight to survive the following winter (Humphries, Thomas, & Speakman, 2002). In yellow‐bellied marmots (*Marmota flaviventris*), for example, years with earlier snowmelt led to an earlier emergence from hibernation and a longer growing season, resulting in an increase in over‐winter survival and reproduction (Ozgul et al., 2010; van Vuren & Armitage, 1991). In Alpine marmots (*Marmota marmota*), earlier snowmelt also led to earlier emergence, but the loss of winter snow cover resulted in a decrease in both litter size and juvenile survival (Rezouki et al., 2016; Tafani, Cohas, Bonenfant, Gaillard, & Allaine, 2013). Thinner snow cover can force marmots to catabolize their fat reserves faster than under the insulation of a deeper snowpack (Tafani et al., 2013), outweighing the benefits of a longer active season. Although buffering against below‐ground freezing temperature can help save on thermoregulatory costs, metabolic costs decrease with decreasing ambient temperature, while arousal costs increase (Geiser & Kenagy, 1988). The extent to which hibernator condition depends on climatic cues is important because even the same climatic variables (e.g., winter snow depth, minimum winter temperatures) can have opposing effects on a given individual, making associated fitness outcomes difficult to predict (Bjorkman, Elmendorf, Beamish, Vellend, & Henry, 2015).

A better understanding of how hibernator phenology is influenced by seasonal environmental conditions and how this may affect fitness will be key in addressing why climate change leads to different fitness outcomes, even in closely related species (Doak & Morris, 2010). Few studies have considered the effects of pertinent season‐specific climatic variables on hibernator phenology and fitness, but a study by Dobson, Lane, Low, and Murie (2016) indicates that a focus on seasonality will help predict how hibernators may respond to warming and increased climate variability in the future.

For instance, snow depth influences soil temperatures and can prevent the ground from freezing (Happold, 1998; Inouye, Barr, Armitage, & Inouye, 2000), which could potentially affect thermoregulatory costs by buffering against below‐ground freezing temperature (as long as they reach the hibernacula). Indeed, while metabolic costs decrease with decreasing ambient temperature during hibernation, arousal costs increase with decreasing ambient temperature (Geiser & Kenagy, 1988). However, this relationship can be complicated by the use of food caches, differing thermal conductance of nests, or differences in individual hibernation energetics that are not related to the gradient between body and soil temperatures (Buck & Barnes, 1999). Although a handful of studies have found that winter conditions can affect fitness up to, and at spring emergence (Rezouki et al., 2016; Tafani et al., 2013; Williams, Buck et al., 2017), the general prediction that below‐freezing hibernacula temperatures should lead to lower body mass and higher mortality rate up to emergence remains extremely difficult to test.

Spring snow dynamics can further affect fitness after emergence because snowmelt determines when food resources become available (Inouye et al., 2000; Ozgul et al., 2010; Rezouki et al., 2016; Vuren & Armitage, 1991; Walker, 1968). Reproduction and subsequent juvenile emergence is generally timed with peak food abundance in hibernators (Walker, 1968), as observed in other taxa (Visser et al., 2006). Hot and dry summers can cause grasses to senesce earlier, while additional precipitation can delay this process (Walker, 1968), affecting whether juvenile emergence is matched with peak food abundance or not.

These seasonal responses are further complicated by demography, since phenological and fitness responses to climate can vary across age classes (Rezouki et al., 2016) and sex (Sheriff, Richter, Buck, & Barnes, 2013). Juveniles have the least amount of time to gain body mass before hibernation, and they may also expend more energy while hibernating due to their large surface‐to‐volume ratio (Kortner & Geiser, 2000; Sherman & Runge, 2002), making them more susceptible to phenological mismatches than older age classes (Miller‐Rushing et al., 2010). In relatively short‐lived species, this may have a large impact on overall population abundance and persistence because population growth is quite sensitive to proportional changes in juvenile survival (Oli, Slade, & Dobson, 2001). In arctic ground squirrels (*Spermophilus parryii*)*,* snow cover affects the emergence of females, while male emergence is more influenced by female emergence (Sheriff, Richter et al., 2013), providing support for the idea that males and females have the potential to be affected differently by specific climatic variables, with consequences for sex‐specific fitness. Indeed, a recent study has shown that female and non‐reproductive male arctic ground squirrels can extend or reenter hibernation to avoid late spring snowstorms, but that plasticity in the use of heterothermy in spring did not extend to reproductive males, who have to end hibernation early to undergo spermatogenesis (Williams, Klaassen et al., 2017).

**Figure 1:**
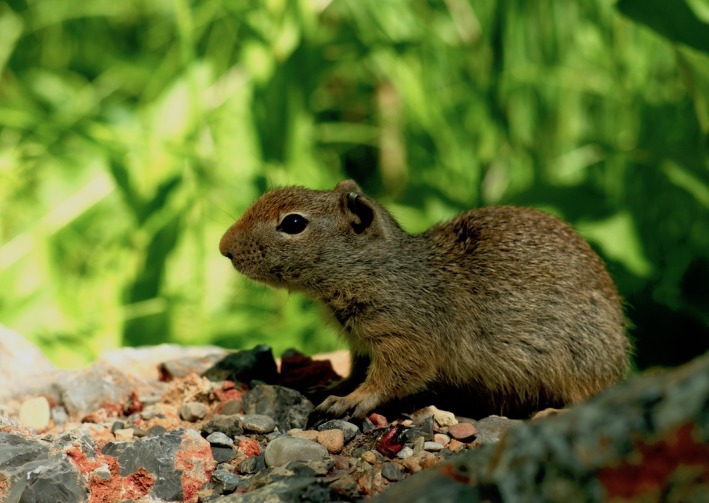
Photo of the Uinta ground squirrel, *Urocitellus armatus*, in Logan Canyon, UT

Because worldwide temperatures are predicted to continue increasing (IPCC, 2013), it is essential to broaden our understanding of how hibernators respond to season‐specific variability in climate. We analyzed mark–recapture data from a population of Uinta ground squirrels (UGS, *Urocitellus armatus*; Figure 1) over a 50‐year period to assess how climate variability affected the survival of a hibernating species endemic to the western U.S. In northern Utah, where the data were collected, temperatures have been increasing (dos Santos, Neale, Rao, & Silva, 2011) and winter snowfall has already decreased by nearly ten percent in the last half‐century, with an increasing proportion of winter precipitation falling as rain instead of snow (Gillies, Wang, & Booth, 2012).

We estimated annual survival using historical (1964–1968) and contemporary (2014–2017) mark–recapture data in an effort to understand how season‐specific climate affected long‐term changes in a key fitness component of the species’ life cycle. We expected that a declining snowpack could cause a decrease in survival, especially for juveniles, or an increase in survival if snow recedes sooner in the spring, leading to earlier availability of forage that would benefit all age classes. We also predicted that warmer summers may cause food resources (i.e., grasses) to senesce earlier, leading to a shortage of food before estivation that would have a negative impact on UGS over‐winter survival, particularly in juveniles. By examining annual changes in survival as they relate to key phenological events in the UGS life cycle (emergence, reproduction, juvenile emergence, and estivation), we hope to determine whether survival has changed over the last 50 years in response to warming, and pinpoint which of the season‐specific climatic and phenological variables may affect this key vital rate across age‐ and sex‐classes.

## 2 METHODS

### 2.1 Uinta ground squirrel ecology

Uinta ground squirrels typically emerge from hibernation between March and April and enter estivation by August, depending on elevation, age, and sex (Eshelman & Sonnemann, 2000). UGS mate within a week of emergence, and adult and yearling females can produce a single litter each year of four to six juveniles (Eshelman & Sonnemann, 2000). Maximum life expectancy is 7 years in the wild, but average life expectancy for juveniles is approximately 1.46 years (Oli et al., 2001). Yearling males rarely emerged from hibernation in breeding condition, while the number and sequence of emergence of females in the early spring affected breeding and social organization by limiting the number of females which retained residences in the study area (Paul, 1977; Walker, 1968). Spring climatic conditions influence emergence date and emergence order of each sex and age class: adult males and females emerge before yearlings in warmer springs, but colder springs cause a more synchronous delayed emergence of sex and age classes (Knopf, 1973), making it more likely for yearling females to breed (Walker, 1968). The diet of UGS is composed of graminoids and forbs, and historically they primarily consumed Kentucky bluegrass (*Poa pratensis*; Walker, 1968). Contemporary surveys show that the site is now dominated by cheatgrass (*Bromus tectorum*), but *Poa *spp. including *Poa pratensis* are still present (C. Falvo, unpublished data). Extensive data were collected from 1964 to 1971 on UGS behavior and demography (Knopf & Balph, 1977; Slade & Balph, 1974; Walker, 1968), but in 1968 the population was reduced by approximately half to study density‐dependent dynamics (Slade & Balph, 1974); thus, we excluded those later years from our analysis. Contemporary data collection began in 2014 up to 2017 in the same location, in order to make comparisons to historical survival probabilities.

### 2.2 Field site and data collection

The contemporary data were collected from 2014 to 2017 at the Utah State University Forestry Field Station in Logan Canyon, UT, in accordance with IACUC protocol #2220. Sites were checked approximately every 3–6 days starting in March for signs of emergence, and there were 9 capture dates in 2014, 12 in 2015, 19 in 2016, and 23 in 2017. There were 95 unique captures in 2014, 55 in 2015, 86 in 2016, and 44 in 2017. Historical data from 1964 to 1968 were collected in the same location by previous researchers detailing the behavior, social, and population dynamics of UGS. Historical data collection was considered a census since technicians were present at the site the entire summer, from emergence until estivation (i.e., prolonged torpor during a hot period; UGS transition to estivation from hibernation without emerging from their burrows; for more detailed description of site and capture methods, see Knopf & Balph, 1977; Slade & Balph, 1974; Walker, 1968).

UGS were caught using Tomahawk live‐traps (Tomahawk, Wisconsin, USA) that were baited with rolled oats and peanut butter. Traps were set early in the morning before squirrels were active, checked within an hour, and squirrels were processed within 2–3 hr of capture. At initial capture, the age of the animal was determined and individuals were classified as juveniles (young of the year), yearling (1‐year‐old), or adult (≥2‐year‐old) based primarily on body mass, reproductive status, and comparison with individuals of known age from previous years of trapping. Each individual's mass was determined by placing the animal in a cloth bag and using a Pesola scale (±2 g.; Pesola Company, Baar, Switzerland). For contemporary data, additional morphometric variables (body, hind foot, and tail length) were measured using a measuring tape or calipers. The reproductive status of males and females was recorded (males as scrotal or non‐scrotal; females as pregnant, lactating, or non‐reproductive). Small, numbered, noncorrosive metal ear tags (Monel 1005‐1, National Band and Tag Company, Newport, Kentucky) were placed on each squirrel at first capture for identification upon recapture. Passive Integrative Transponders (PIT tags, Biomark) were also injected under the skin to keep track of individuals in case of ear tag loss.

### 2.3 Impact of season‐specific climate on UGS viability fitness

Climate data were obtained from a weather station approximately 26 km (16 miles) from the field site. Although a weather station is located <1 km from the field site, data were only available from 2009 to present. Climate variables of interest were compared between these two weather stations for available co‐occurring years and were found to be highly correlated (see Appendix A).

#### 2.3.1 Fall

Snow acts as an insulator and can prevent the ground from freezing (Happold, 1998; Inouye et al., 2000). In Alpine marmots, when snow depth is >110 cm, the burrow temperatures are thought to be buffered against air temperatures (Tafani et al., 2013, Appendix D). To assess the potential influence of below‐freezing temperatures on UGS survival before an insulating layer of snow was present, the number of days below freezing that occurred after estivation began and before permanent snow cover occurred (August–November or December) were tallied up each year *t* (e.g., August to December 2015), and the impact of that variable on survival was assessed between year *t* and *t* + 1.

#### 2.3.2 Winter

Because winter conditions could affect over‐winter survival as well as fitness postemergence, we considered the average snow depth and temperature from December of the previous year (*t *− 1) until emergence the next spring (*t*) in examining the influence of winter conditions on survival from *t* − 1 to *t* (direct effect) and from *t* to *t* + 1 (cross‐seasonal effect).

#### 2.3.3 Spring

Snowmelt determines when food resources become available (Inouye et al., 2000; Rezouki et al., 2016; Vuren & Armitage, 1991; Walker, 1968). In years when squirrels emerged long before snowmelt (snow melted late relative to other years), their body mass typically declined before increasing again as food became accessible (Knopf & Balph, 1977), indicating the importance of spring temperatures as they relate to forage availability and body mass at spring emergence. Since temperatures in year *t* could influence survival from *t* − 1 to *t* or from *t* to *t* + 1, we considered the maximum temperatures between March 15th and March 31st (per Knopf & Balph, 1977) in our model selection framework.

#### 2.3.4 Summer

Summer precipitation and temperatures influence food availability, with hot and dry summers causing earlier plant senescence (Walker, 1968). Precipitation may be positively correlated with survival through improved forage quality (Armitage, 1994). Previous studies have shown that timing of UGS estivation roughly coincides with the drying out of vegetation (Knopf, 1973), suggesting declines in precipitation have the potential to shorten the active season via changes in plant availability. To examine the influence of summer food conditions in relation to UGS phenology, growing degree days (at 0°C; Frank, 1996) were calculated from emergence until the estimated date of juvenile emergence, when forage quality and quantity is essential to this age class. We also considered an aridity index that incorporates average temperature and precipitation from June to August (BGI, Bagnouls‐Gaussen Index: daily rainfall (mm) minus twice the average temperature (°C) (Canale, Ozgul, Allainé, & Cohas, 2016; Aridity Index, 2014), as well as average temperature and average precipitation alone as variables that could influence survival to the following year.

### 2.4 Capture–mark–recapture analyses and model selection

To determine whether UGS survival has changed over time, we used Cormack–Jolly–Seber (CJS, Lebreton, Burnham, Clobert, & Anderson, 1992) capture–mark–recapture (CMR) models developed in R (R Core Team, 2013, version 3.2.3) using the RMark package (Laake, 2013) to estimate annual apparent survival (*ϕ*) and recapture probability (*p*) from both historical and contemporary data.

#### 2.4.1 Goodness‐of‐Fit

We used goodness‐of‐fit (GOF) tests computed in the RELEASE software implemented in program MARK (Cooch & White, 2006), which tests assumptions of the CJS model that every marked animal present at time *t* has the same probability of recapture, and that every marked animal immediately after *t* has the same probability of surviving to *t* + 1 (White & Cooch, 2012) (see Appendix B for details). We considered a global model where survival was allowed to vary by age, sex, and time period (historical vs. contemporary). Although a global model may pass GOF tests, this does not imply that assumptions of the CMR modeling framework are met perfectly. We therefore estimated $\hat{c}$ to adjust model likelihoods for over‐dispersion in the data by dividing the overall *χ* ^2^ values by the total degrees of freedom, which is not biased as high as the median $\hat{c}$ approach for CJS models (White, 2002).

#### 2.4.2 Detection probability (*p*)

Because of a 46‐year gap in the CMR data, appropriate recapture and survival parameters were fixed to 0 between the historical and contemporary study periods. Additionally, data on adult males were not available during the first 2 years of the historical study, and thus, these parameters were also fixed to 0. For historical years, we fixed *p* to 1 because trapping was considered a census in those years (Slade & Balph, 1974). Since estimates for *p* consistently converged to 1 for females, and for both sexes in 2016, we also fixed *p* = 1 for those sex‐year combinations. All other recapture probabilities were estimated by comparing models that allowed variability by age, sex, and year using QAICc (AIC adjusted for sample size and over‐dispersion; Burnham & Anderson, 2002). The top performing model for *p* was then used alongside alternative parameterizations for *ϕ*.

#### 2.4.3 Apparent survival probability (*ϕ*)

To keep our model selection process down to a reasonable number of models (Burnham & Anderson, 2002), we first examined the influence of demographic categories and time (age, sex, and year) on apparent survival. We carried forward the highest‐ranked term(s) into a final set of models where we considered univariate effects of phenological variables and seasonal climate variables (emergence dates, season length; winter, spring, summer, or fall climate) interacting with age. By avoiding model dredging, we acknowledge we may have overlooked certain variable combinations supported by the data, and of importance in affecting actual UGS survival.

1. *Demographic categories*. Within each subset of models, we only considered models that tested for ecological hypotheses of interest. For instance, we expected survival to be different between males and females because females bear the cost of reproduction, and after gestation and provisioning for their offspring, they have less time to gain mass before estivation begins (Knopf, 1973). We also anticipated differences in survival between juveniles and older age classes because juveniles have the shortest amount of time to gain mass prior to estivation. Models with three age classes (juvenile, yearling, and adult) vs. two age classes (juvenile, yearling, and adult combined) were compared, as we did not anticipate large differences in survival between yearlings and adults (Oli et al., 2001).
2. *Phenological variables*. We compared models that accounted for the effect of emergence date, season length, and a cross‐seasonal effect of either on survival (*t *− 1 to *t* and *t* to *t *+ 1). Emergence date was the calendar date the first squirrel was seen in a given year, and season length was based on the amount of time squirrels were known to be active, from emergence to estivation in a given year. An early emergence date may be beneficial (if forage availability and snowmelt are also initiated early), or detrimental (if snow cover persists beyond emergence). Similarly, a longer season may be beneficial if food resources have not yet senesced, and nutritional quality is not compromised.
3. *Seasonal climate variables*. We considered the number of below‐freezing days after estivation and before permanent snowpack, winter temperature, winter snowfall, maximum March temperature, growing degree days (GDD), Bagnouls‐Gaussen summer drought index (BGI), summer temperature, and summer precipitation.

#### 2.4.4 Model selection

We used QAICc to score model(s) in each tier of our model selection process (see above), while ensuring that each model reflected ecological hypotheses of interest, as per Lebreton et al. (1992). Because time‐varying climate variables are redundant with annual changes in survival, generic (fixed effect) time variation in survival (if selected in our demographic set of models) was never considered along with time‐varying covariates in the final model set. Overall, this tiered approach was preferred because it allowed us to consider models that reflected relevant ecological hypotheses while restricting the number of models considered to a reasonable level.

To evaluate how well our best‐performing models fit the data, we calculated *R*, the ratio of deviance reduction for a model relative to a fully saturated model and a null model (Iles et al., 2013). This involved calculating the deviance reduction measure (*D*_MOI_ = 1 − dev_MOI_/dev_NULL_) where MOI is the model of interest and NULL is the least parameterized model that was considered (~age class). This value was used to calculate the ratio of deviance reduction (*R* = *D*_MOI_/*D*_FULL_) where FULL refers to a fully saturated temporal model (~ age class * time), providing a relative measure of deviance reduction.

## 3 RESULTS

### 3.1 Trends in climate and phenological variables

**Figure 2:**
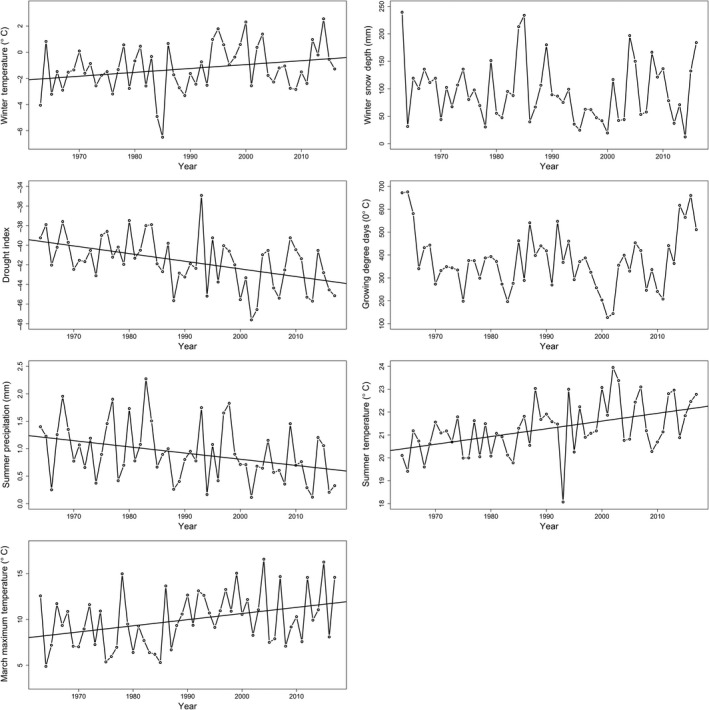
Trends in climate variables (winter temperature, winter snow depth, BG drought index, growing degree days, summer precipitation, summer temperature, and maximum March temperature) from 1960 to 2017 in Logan, UT

Trends in the climate variables recorded at the Utah State University weather station that we considered in our analysis indicated warming over the study period, consistent with global and regional trends (IPCC, 2013; Figure 2). Specifically, average winter temperatures marginally increased over time (by 1.58°C; *p* = 0.054, Adj. *R* ^2^ = 0.051), and maximum temperatures in March (March 15–31) have increased significantly over time (by 3.56°C; *p = *0.007, Adj. *R* ^2^ = 0.111). Unlike regional trends (i.e., the state of Utah), average winter snow depth did not decline at the local weather station (*p* = 0.435, Adj. *R* ^2^ = −0.007). Average summer temperatures also increased (1.79°C; *p* < 0.001, Adj. *R* ^2^ = 0.199), while average summer precipitation declined over the study period (−0.60 mm; *p* = 0.012, Adj. *R* ^2^ = 0.097). Although the BG drought index declined over time (−4.14; *p* < 0.001, Adj. *R* ^2^ = 0.201), indicating drier summer conditions in recent years, growing degree days (from average emergence to average juvenile emergence from their natal burrow) did not significantly change over time (*p* = 0.692, Adj. *R* ^2^ = −0.016). Phenological variables did not exhibit any temporal trend (emergence: Welch's *t* test, *t = *0.0258, *df* = 5.5412, *p* = 0.9803; season length: *t* = 0.9381, *df* = 6.4662, *p* = 0.3819): the average emergence dates (historical calendar date = 92.4, contemporary Julian date = 92.3) and season length (historical = 128.6 days, contemporary = 123.4 days) did not change over time either.

### 3.2 Capture–mark–recapture analyses

The GOF test conducted on the most parameterized model indicated that juveniles in 1967 violated the assumptions of Test 3 (*χ* ^2^ = 74.1728, *df* = 19, *p* = 0). However, the addition of cohort effects for these two groups resolved the issue (*χ* ^2^ = 15.6503, *df* = 16, *p* = 0.4776; see Appendix B for details). Note that Test 3 determines whether individuals tagged that particular year were seen again at the same rate as individuals tagged in previous years. Because the survival interval that violated assumptions was the last for our study period, we are not concerned about this result which simply indicates that our study ended and was reinitiated many years later. We estimated a $\hat{c}$ value of 3.89 and used it to correct for moderate over‐dispersion in the data.

The top model for detection probability included an effect of sex and outperformed the next best model by 2.001 QAICc point (*p* ~ time). We retained this parameterization for *p* for the remainder of the model selection process. There were several models within 2 QAICc points that explained variation in apparent survival probability among years and demographic categories, but all top models included age class (juveniles vs. adults and yearlings combined); hence, this term was included in the following tiers of model selection. On average, adult and yearling survival was higher (estimate = 0.41 ± 0.01) than juvenile survival (estimate = 0.29 ± 0.01).

In an effort to reduce the overall number of models considered in our candidate set, we explored trends in the relationship between age and sex‐specific annual survival estimates and each climate variable of interest using linear regression. Climate regression coefficients that achieved less than the liberal significance threshold of *p* < 0.15 were retained for further analyses. Following this screening process, we retained a subset of climate variables: winter temperature, snow depth, maximum March temperature, and season length, and considered full and partial interactions with age class in the CMR analyses when they made biological sense (Table 1).

**Table 1:** Final model selection, where “age” refers to the age class (juvenile vs. adult and yearling), “ay” and “j” are used for models with partial interactions, “wint” refers to winter temperatures, “marm” refers to March maximum temperature, and “snow” refers to snow depth. Number of parameters (np), adjusted AIC (QAICc), change in QAICc relative to the top model (ΔQAICc), weight of each model (Wt), deviance (QDeviance), and relative reduction in deviance (*R*) are also presented. We present the parameterization associated with the survival probability *ϕ* while the best parameterization for *p* is maintained

Model	Np	QAICc	ΔQAICc	Wt	QDeviance	*R*
~age * wint	5	1782.88	0.00	0.29	788.49	0.277
~ay +j + j:wint	4	1783.42	0.53	0.22	791.03	0.172
~j + ay + ay:wint	4	1785.09	2.20	0.10	792.70	0.102
~j + ay + ay:marm	4	1785.32	2.44	0.09	792.93	0.092
~age	3	1785.54	2.66	0.08	795.16	0.000
~j + ay + ay:snow	4	1785.60	2.72	0.07	793.21	0.081
~ay + j + j:snow	4	1786.79	3.90	0.04	794.40	0.032
~age * snow	5	1786.86	6.29	0.04	792.46	0.112
~age * marm	5	1787.07	7.48	0.04	792.67	0.103
~ay + j + j:marm	4	1787.31	7.55	0.03	794.92	0.010
~1	2	1799.84	7.76	0.00	811.46	NA

**Figure 3:**
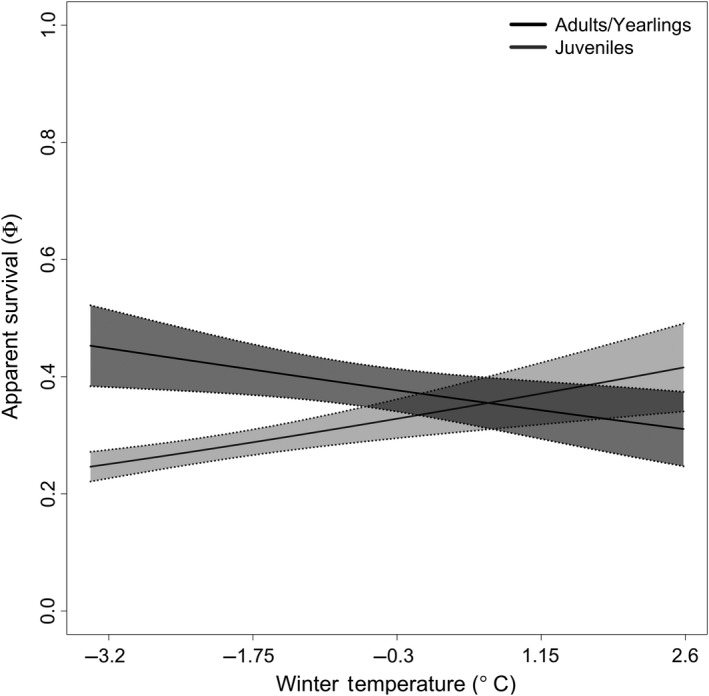
The interactive effect of age class (adults and yearlings = black, juveniles = gray) and winter temperature on apparent survival (*ϕ*) from the top‐ranked model

**Figure 4:**
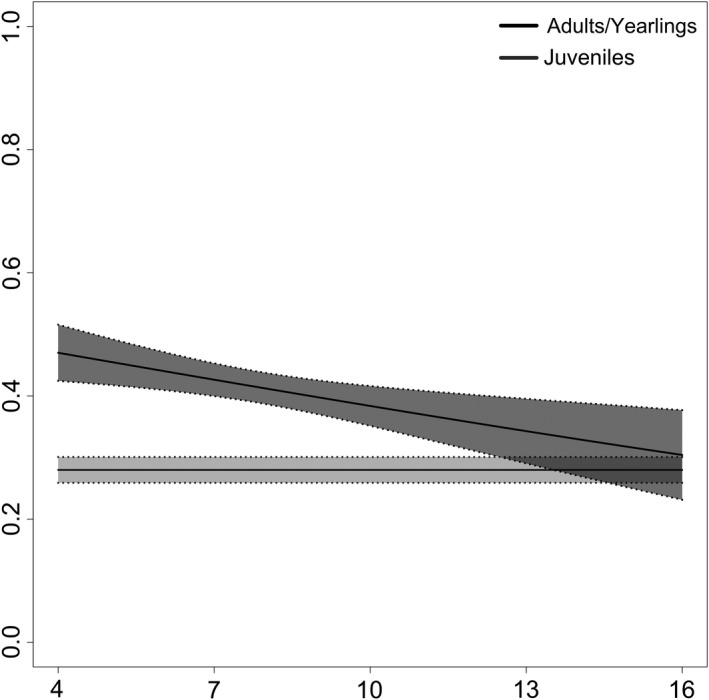
The effect of age class (adults and yearlings = black, juveniles = gray), March maximum temperature, and the partial interaction between adults/yearlings on apparent survival (*ϕ*) from the 4th ranked model.

The top model included a full interaction between age class (juveniles, yearlings/adults) and winter temperatures (Table 2 and Figure 3). The second model (ΔQAICc = 0.53) included a partial interaction between juveniles only and winter temperatures. Both of these models suggest that a warmer winter is beneficial for juvenile survival, while the best‐performing model further suggests that warmer winters are associated with a decrease in adult and yearling survival. Models that followed closely in likelihood included a partial interaction between adults/yearlings and winter temperatures (ΔQAICc = 2.20), and a partial interaction between adults/yearlings and March maximum temperature (ΔQAICc = 2.44), which indicated that hotter March temperatures had negative effects on adult/yearling survival (Figure 4).

**Table 2:** Beta survival estimates from the top model, which includes an interaction between age (adult/yearling, juvenile) and winter temperature, along with standard error (*SE*), lower confidence limit (LCL), and upper confidence limit (UCL)

Group	Beta estimate	*SE*	LCL	UCL
Phi: j	−0.8540	0.0578	−0.9673	−0.7408
Phi: ay	−0.3956	0.0557	−0.5047	−0.2865
Phi: wint	0.2400	0.0594	0.1235	0.3565
Phi: ay:wint	−0.4275	0.0854	−0.5949	−0.2601
*p*	0.6332	0.5395	−0.4242	1.6906

## 4 DISCUSSION

### 4.1 Does climate play a role in explaining observed variation in survival?

Although temperatures at the study site increased in both summers and winters over time, and precipitation in summer declined over the span of this study (as expected from larger regional trends), snow depth at our site did not significantly change. Snow depth did not appear to be an important variable in any of the survival models. Interestingly, phenological variables (emergence and season length) did not indicate a significantly linear change over time either, despite changes in climate. However, it would be difficult to detect a linear change in emergence phenology from only 7 data points spread over 50 years. Hence, the importance of long‐term study in addressing the role climate change plays in shifting phenologies and other key life history traits (Clutton‐Brock & Sheldon, 2010).

Below‐freezing winter temperatures are associated with increased arousal costs but substantial energy savings due to a decreased cost of thermoregulation, limiting energy expenditure (Geiser & Kenagy, 1988). Overall, one would still expect the cost of hibernation (i.e., increased catabolization of fat reserves) to prevail under such conditions, unless snowpack insulation is sufficient to create a buffer (Tafani et al., 2013). Relying on few data points, our results suggest that juvenile survival responds differently to winter temperatures compared to yearlings/adults (Table 2 and Figure 3). It seems that colder ambient temperatures (which strongly correlate with snow pack in our system; correlation coefficient = 0.895, *p* = 0.001) convey a survival benefit to yearlings and adults, but a survival cost to juveniles. In other words, “lower” hibernacula temperatures only decrease the cost of thermoregulation in yearlings and adults, but not in juveniles. Juveniles have a larger surface‐to‐volume ratio than yearling and adults, likely forcing them to expend more energy while hibernating (Kortner & Geiser, 2000; Sherman & Runge, 2002); this could explain why they tend to survive over‐winter at a higher rate when ambient winter temperatures are above freezing. Social dynamics within hibernacula, timing of immergence (i.e., juvenile typically enter estivation 2–3 weeks after older age classes; Knopf, 1973) and hibernacula depth (Knopf & Balph, 1977) could also explain observed differences in juvenile survival when compared to older age classes. On the other hand, colder temperatures could benefit adults/yearling UGS as they can reduce the energetic output necessary during hibernation (Kronfeld‐Schor & Dayan, 2013) and allow UGS to remain in hibernation longer (Turbill & Prior, 2016).

Maximum temperatures in March (March 15–31) have increased significantly over the span of this study (by 3.56°C; *p = *0.007, Adj. *R* ^2^ = 0.111). Hotter March temperatures did not have an effect on juvenile survival but did have a negative effect on adult/yearling survival (Table 3). The hotter temperatures at this time could be related to earlier snowmelt and the start of spring green‐up of vegetation. Previous research on the same population found that in early springs, more UGS females failed to produce litters or disappeared entirely prior to recruitment (Walker, 1968), which supports our results for adult and yearling UGS. Earlier springs led to asynchronous emergence across age classes, with adults emerging earlier than yearlings. When this happens, adults act more aggressively toward yearlings, since they are more likely to have already established territories and mated, which often results in yearlings failing to reproduce or emigrating away from their natal colony (Paul, 1977; Walker, 1968). Earlier emergence may also expose UGS to predation for a longer period of time. In other hibernating species, a later emergence led to increased survival since remaining in hibernation provided protection from predation by most predators (Bieber, Lebl, Stalder, Geiser, & Ruf, 2014; Bryant & Page, 2005; Turbill, Bieber, & Ruf, 2011), with the exception of burrowing predators such as badgers (*Taxidea taxus*) and weasels (*Mustela *spp.) (Walker, 1968). Although badgers are present at our site, hawks (e.g., *Buteo jamaicensis*), foxes (*Vulpes vulpes*), and coyotes (*Canus latrans*) are also known predators of UGS (Amend, 1970).

**Table 3:** Beta survival estimates from the 4th ranked model, which includes an effect of age, March maximum temperatures, and a partial interaction between adults/yearlings (ay) and March maximum temperatures, along with standard error (*SE*), lower confidence limit (LCL), and upper confidence limit (UCL)

Group	Beta estimate	*SE*	LCL	UCL
Phi:j	−0.9449	0.0532	−1.0491	−0.8407
Phi:ay	−0.3008	0.0551	−0.4089	−0.1928
Phi:ay:marm	−0.2877	0.0991	−0.4819	−0.0934
*p*	1.2420	0.6557	−0.0433	2.5272

Although summer climate variables did not rank in our model selection, we had expected hotter and drier summers to have a negative impact on the quality of food or cause estivation to begin earlier. In 1966, the warmest and driest year within the historical study, the active season ended the earliest and was the shortest (even though emergence from hibernation that year was intermediate to the other years), while the cooler and rainier years had seasons that ended later (Knopf, 1973). In UGS, juvenile emergence from their natal burrows typically coincides with peak food abundance (Walker, 1968), and growing degree days can reflect the phenology of food resources. The presence of cheatgrass at the study site (C. Falvo, unpublished data), that was never mentioned to be present historically (Walker, 1968), may be increasing forage availability to UGS by outcompeting sagebrush (Stewart & Hull, 1949). Sagebrush is only consumed in small amounts by UGS (~10%) and is not their preferred food (Walker, 1968)). Juveniles emerge at a time when grasses are most abundant (Walker, 1968), further suggesting that juveniles do not eat large amounts of sagebrush. The substitution of sagebrush by cheatgrass may have improved forage availability for juveniles, particularly via massive seed production, and continued warming may allow cheatgrass to further dominate the area (Chambers, Roundy, Blank, Meyer, & Whittaker, 2007; Compagnoni & Adler, 2014) and benefit this particular UGS population unless it alters the fire regime (as it does at lower elevations; Knapp, 1996). Cheatgrass establishment, biomass, and seed production are most strongly constrained by cold temperatures (Chambers et al., 2007), further suggesting that warming temperatures benefit its establishment and persistence. The increase in cheatgrass biomass which persists through the summer at high elevations within its range could outweigh the negative effects of a decline in native grasses for UGS. However, the only years in this study with warmer than average summers were 2016 and 2017. Although we did not detect any changes in the number of growing degree days over the last 50 years, overall summer temperatures are predicted to continue warming; it will be interesting to assess whether cheatgrass can buffer UGS against the predicted negative impacts of hotter and drier summer in the future.

**Figure 5:**
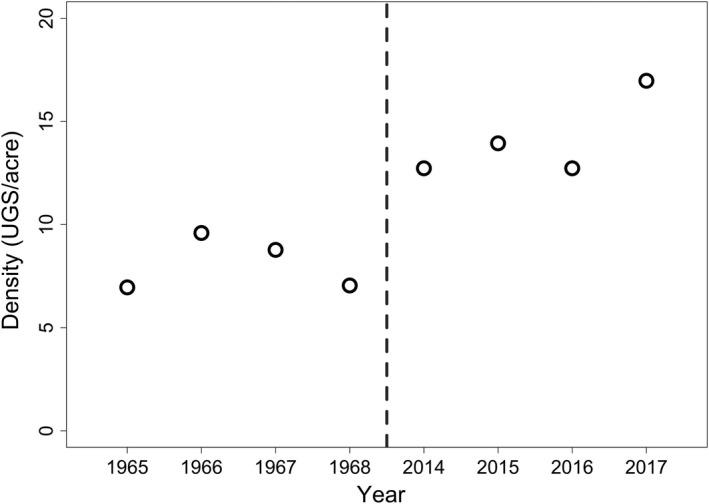
Density of UGS per acre over years of the study

Although the historical capture area was more heterogeneous in its vegetation than it currently is, and the trapping area today might not be exactly in line with what it used to be, we used the Horvitz–Thompson estimator (Horvitz & Thompson, 1952) to calculate approximate density (Figure 5). Since juveniles are more likely to emigrate, density was only calculated for adults and yearlings (i.e., residents). We observed an increase in population density during that time period (Figure 5), suggesting resources are less limited today than they used to be. This supports the idea that cheatgrass, which now dominates the study site, may provide a better food source to UGS than native plants did historically.

## 5 CONCLUSIONS

These results add to our understanding of the relationship between year‐to‐year climate variability, phenology, and demography, as illustrated by changes in survival in hibernating ground squirrels. Although the phenology of UGS does not appear to have changed over time, UGS survival is affected by climate, with each age class responding differently. Warmer winter temperatures may increase juvenile survival, but decrease adult survival, making it difficult to predict how this population may respond to prolonged and increasing warming. We suspect warmer temperatures in our study area have facilitated the invasion of cheatgrass, and that this relatively new food source is benefiting UGS, especially juveniles whose emergence coincides with leaf and seed production, which may explain the observed increase in UGS density over time. Our results suggest that the potential negative impact of warming on yearling/adult UGS survival seems outweighed by the observed increase in juvenile survival fueled by warmer winter temperatures and cheatgrass invasion. Additional work will help assess whether these population‐level benefits will continue, or will remain short‐lived in light of continued warming (Rezouki et al., 2016; Tafani et al., 2013).

It is, however, impossible to infer from our results how seasonal changes in climate could affect population structure and dynamics without a thorough understanding of the impact climate variability has on fertility components such as age at maturity, reproductive success, and age at last reproduction. Indeed, after an experimental reduction in UGS population density back in the 1960s, age at maturity and fertility contributed most to changes in the population growth rate in lawn and edge habitats (Oli et al., 2001). However, both survival and fertility rates contributed equally to proportional changes in population growth rate within non‐lawn habitats. Future studies could help establish a solid connection between climate variability and UGS population dynamics by focusing on seasonal climate impacts on fertility and density estimates across habitats types, but this would involve manpower and resources that were not available at the time of our study.

## CONFLICT OF INTEREST

None declared.

## AUTHOR CONTRIBUTION

Caylee Falvo was responsible for the design of the study, collection of data, analysis and interpretation of the data, and writing of the manuscript. David Koons was responsible for analysis and interpretation of the data and editing of the manuscript. Lise Aubry was responsible for design and supervision of the study, analysis and interpretation of the data, and writing of the manuscript.

## Figures and Tables

**Figure A1:**
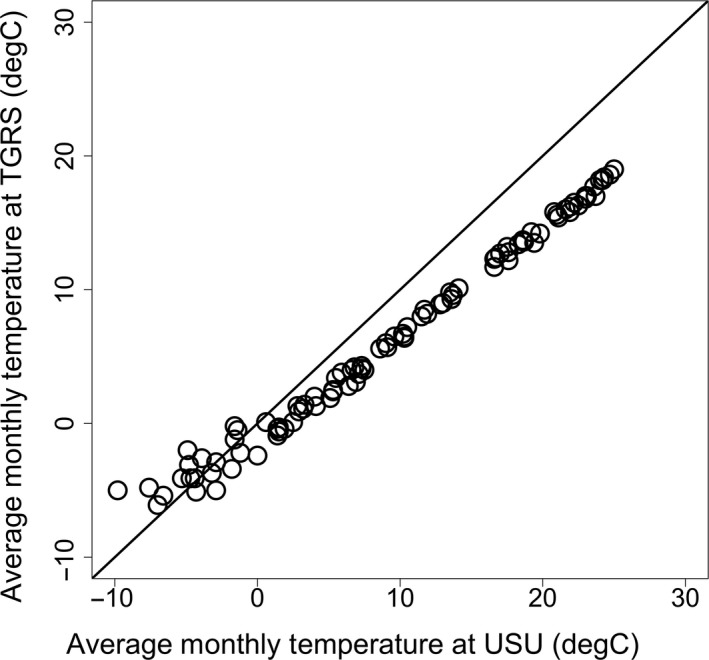
Relationship between TGRS and USU average monthly temperatures from 2009 to 2016 (*R* ^2^ = 0.9882; *p*‐value <0.001; Pearson product‐moment correlation = 0.994)

**Figure A2:**
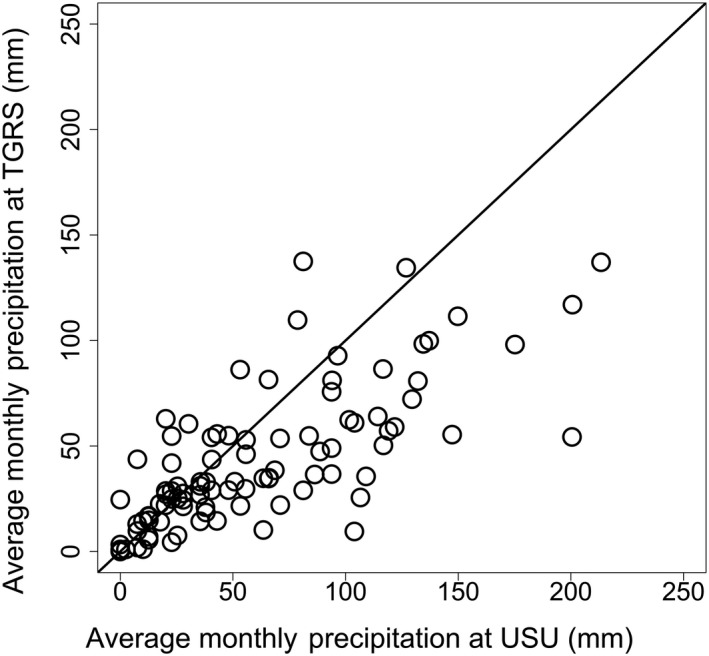
Relationship between TGRS and USU average monthly precipitation from 2009 to 2016. Dotted line indicates the strength of the association between both weather stations, USU and TGRS, respectively (*R* ^2^ = 0.5445; *p*‐value <0.001; Pearson product‐moment correlation = 0.738)

## Data Availability

Mark‐recapture data and associated code are available from Dryad (https://doi.org/10.5061/dryad.m29k410).

## APPENDIX A

We present correlation coefficients between monthly temperature and precipitation for two weather stations: the Tony Grove Road weather station, located <1 mile from the study site, for which only climatic data from 2009 onwards are available, and the Utah State University weather station located 17 miles from the study area, for which climatic data are available since 1893.

### WEATHER STATION INFORMATION

Tony Grove Snowtel (TGRS): located in Logan Canyon, Cache County, Utah, 0.9 miles from our field site. Elevation: 6,332 feet; latitude 41.89; Longitude: −111.57. Reporting since 2009‐10‐01 (Source: Utah State University Climate Center; https://climate.usurf.usu.edu/mapGUI; site accessed: September 9th 2017).

Utah State University (USU): located in Logan, Cache County, Utah. Elevation: 4,778 feet; latitude: 41.75; longitude: −111.8, located 15.96 mi (25.69 km) from our field site. Reporting since 1893‐01‐01 (Source: Utah State University Climate Center; https://climate.usurf.usu.edu/mapGUI; site accessed: September 9th 2017).

## APPENDIX B Goodness‐of‐fit tests and estimating median c‐hat

### GOODNESS OF FIT

We used goodness‐of‐fit tests computed in the RELEASE software implemented in program MARK, which tests the assumptions of the CJS model that every marked animal present at time *t* has the same probability of recapture, and that every marked animal immediately after *t* has the same probability of surviving until *t* + 1 (Cooch & White 2006). We considered a global model where survival was allowed to vary by age, sex, and time period (historical vs. contemporary) to test these assumptions; results are presented in Table B1. The null hypothesis is that the assumptions of the CJS model are true so rejecting the null indicates a violation of assumptions. The GOF test conducted on this model indicated that juveniles in 1967 violated the assumptions of Test 3 (*χ* ^2 ^= 74.1728, *df* = 19, *p* = 0). However, the addition of cohort effects for these two groups resolved the issue (Table B2; *χ* ^2 ^= 15.6503, *df* = 16, *p* = 0.4776). Test 3 determines whether individuals tagged that particular year are seen again at the same rate as individuals tagged in previous years, and the survival interval that violated assumptions was the last for our study period, so we are not concerned about this violation which simply results from the fact that our study ended.

### MEDIAN $\hat{c}$

We estimated the median c‐hat to adjust model likelihoods for over‐dispersion in the data by dividing the overall *χ* ^2^ values (74.173) and dividing by the total degrees of freedom (19) to obtain a $\hat{c}$ of 3.90 (Laake, 2013).

**Table B1:** Goodness‐of‐fit results from less parameterized model, Phi ~age + sex + time period, which included cohort effects for juveniles in 1967, where “Iniage” refers to initial age and includes “A/Y” (adults/yearlings) and “J” (juveniles), “Grp” refers to contemporary vs. historical, “Freq” defines known dead recaptures (−1 refers to known deaths), and “Cohort” includes an effect of birth cohort

Group	Iniage	Sex	Grp	Freq	Cohort	*χ* ^2^	*df*	*p*‐Value
1	A/Y	F	C	−1	0	0.000	0	1.000
2	J	F	C	−1	0	0.000	0	1.000
3	A/Y	M	C	−1	0	0.000	0	1.000
4	J	M	C	−1	0	0.000	0	1.000
5	A/Y	F	H	−1	0	1.366	1	0.243
6	J	F	H	−1	0	0.000	0	1.000
7	A/Y	M	H	−1	0	0.000	0	1.000
8	J	M	H	−1	0	1.669	2	0.434
9	A/Y	F	C	1	0	1.932	2	0.381
10	J	F	C	1	0	2.532	2	0.282
11	A/Y	M	C	1	0	0.579	2	0.749
12	J	M	C	1	0	0.897	2	0.639
13	A/Y	F	H	1	0	3.694	4	0.449
14	J	F	H	1	0	26.010	2	0.000
15	A/Y	M	H	1	0	0.000	0	1.000
16	J	M	H	1	0	35.494	2	0.000
17	J	F	H	−1	1	0.000	0	1.000
18	J	M	H	−1	1	0.000	0	1.000
19	J	F	H	1	1	0.000	0	1.000
20	J	M	H	1	1	0.000	0	1.000
Total						74.173	19	0.000

**Table B2:** Goodness‐of‐fit results from model that also included cohort effects for juveniles in 1967, where “Iniage” refers to initial age and includes “A/Y” (adults/yearlings) and “J” (juveniles), “Grp” refers to contemporary vs. historical, “Freq” defines known dead recaptures (−1 refers to known deaths), and “Cohort” includes an effect of birth cohort

Group	Iniage	Sex	Grp	Freq	Cohort	*χ* ^2^	*df*	*p*‐Value
1	A/Y	F	C	−1	0	0.000	0	1.000
2	J	F	C	−1	0	0.000	0	1.000
3	A/Y	M	C	−1	0	0.000	0	1.000
4	J	M	C	−1	0	0.000	0	1.000
5	A/Y	F	H	−1	0	1.366	1	0.243
6	J	F	H	−1	0	0.000	0	1.000
7	A/Y	M	H	−1	0	0.000	0	1.000
8	J	M	H	−1	0	1.669	1	0.196
9	A/Y	F	C	1	0	1.932	2	0.381
10	J	F	C	1	0	2.532	2	0.282
11	A/Y	M	C	1	0	0.579	2	0.749
12	J	M	C	1	0	0.897	2	0.639
13	A/Y	F	H	1	0	3.694	4	0.449
14	J	F	H	1	0	0.000	1	1.000
15	A/Y	M	H	1	0	0.000	0	1.000
16	J	M	H	1	0	2.982	1	0.084
17	A/Y	F	H	−1	1	0.000	0	1.000
18	J	M	H	−1	1	0.000	0	1.000
19	A/Y	F	H	1	1	0.000	0	1.000
20	J	M	H	1	1	0.000	0	1.000
21	A/Y	F	H	−1	1	0.000	0	1.000
22	J	M	H	−1	1	0.000	0	1.000
23	A/Y	F	H	1	1	0.000	0	1.000
24	J	M	H	1	1	0.000	0	1.000
Total						15.650	16	0.478
